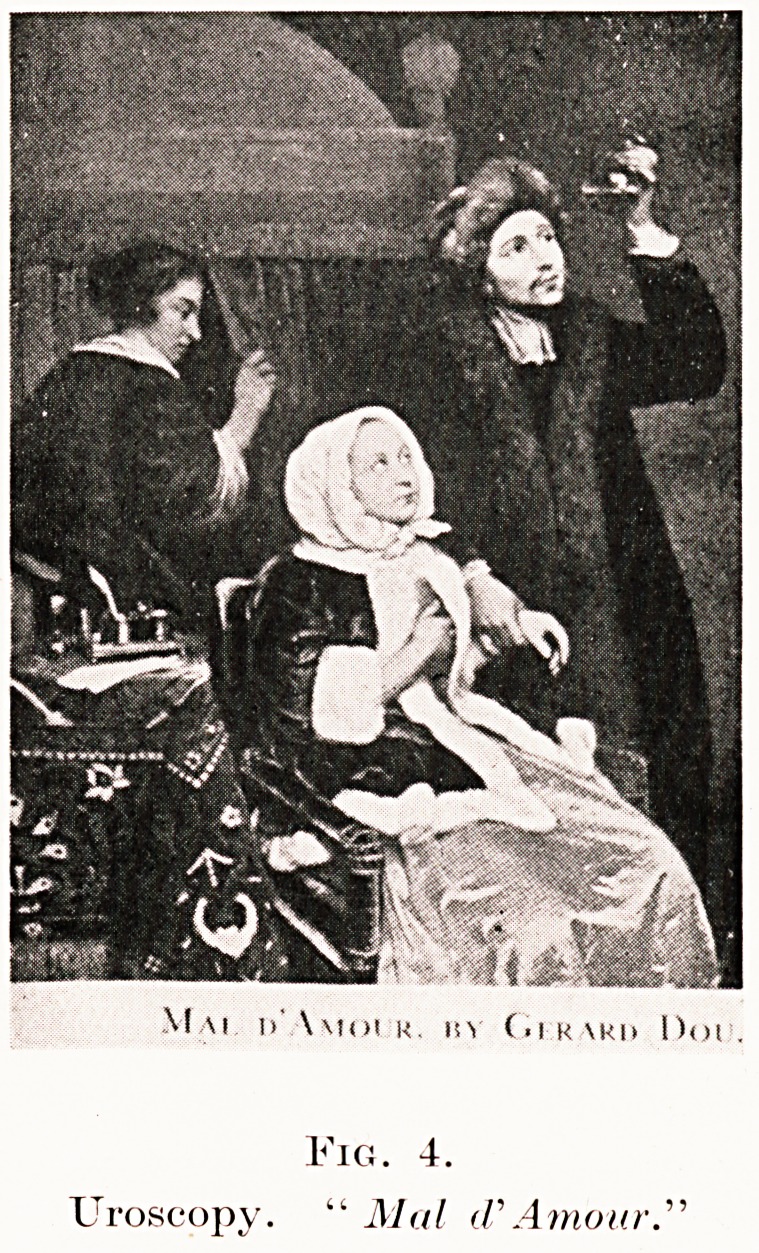# Medicine and the Drama

**Published:** 1939

**Authors:** Leonard A. Moore

**Affiliations:** Honorary Anæsthetist to the Bristol Royal Infirmary


**The Bristol Medico-Chirurgical Journal**

*" Scire est nescire, nisi id me Scire alius sciret."*

SPRING, 1939.

### MEDICINE AND THE DRAMA.

The Presidential Address, delivered on 12th October, 1938, at the opening of the Sixty-sixth Session of the Bristol Medico-Chirurgical Society.

BY

LEONARD A. MOORE, M.B., CH.B.,

*Honorary Anæsthetist to the Bristol Royal Infirmary.*

---

I HAVE chosen the subject "Medicine and the Drama," for two reasons: first, the drama seems to be a stepping-stone to the Presidency of this Society. As you know, the Medical Dramatic Club is the oldest surviving dramatic club in Bristol, and a large number of our presidents have passed through its ranks. Secondly, the drama is the one subject outside our professional interests that can claim a big majority of our members as its devotees.

Medicine in one or other of its varied aspects has been the theme of innumerable plays. Shakespeare alone has five hundred references, and many a play has a clever character-sketch of a doctor either as hero, villain, or comedian. The authors treat us very much as they do the stage parson, exaggerating our peculiarities and minimizing our virtues.

The only play that mentions a contemporary doctor by name is Farquhar's *Twin Rivals* (1702).

> "Does the silly creature imagine that any man would come near her unless it were Dr. Chamberlen."

In preparing this address I have come across extracts from plays that have seemed very apropos of several of the papers we have had read to us lately. Take the latest, Mr. Willway's paper on the brain; in Macbeth we read:—

> *Macbeth:* ". . . The times have been,
> That, when the brains were out, the man would die,
> And there an end; But now they rise again."
>
> *Macbeth,* III. iv.

Dr. Ling's: "The Emotional factor in Disease":—

> "As the mind has a great influence over the body, and is very often the cause of sickness, my custom is first to cure the mind before I proceed to the body . . . her illness proceeds only from a disordered imagination, and a depraved desire to be married."
>
> Molière, "*Love is the Best Doctor.*"

> *King Henry:* "Then you perceive the body of our kingdom,
> How foul it is; what rank diseases grow,
> And with what danger, near the heart of it.
>
> *Warwick:* "It is but as a body, yet, distemper'd,
> Which to his former strength may be restor'd,
> With good advice and little medicine."
>
> *Henry IV* (2), III. i.

As both these extracts were written over three hundred years ago, it appears we have been rather tardy in starting a psycho-therapy department in Bristol.

You all remember Dr. Foss's recent paper on hormone activity which seemed to bring to us old 'uns a vision of renewed vigour and perpetual youth.

Sir Kenelm Digby had a remedy called "Viper Wine," a decoction much in vogue with the superannuated Lotharios of the seventeenth century, the predecessors of the "gay old dogs" of to-day who submit to monkey gland treatment or Steinach operations, or who will (I suppose) in future demand hormone injections.

> "Your Viper wine,
> So much in practice with grey headed gallants,
> But Vappa to the nectar of her lips."
>
> Messinger, *Believe As You List.*

Vappa was a pallid wine, an innocuous drink: the lady evidently was not so innocuous. The viper was chosen, I suppose, because it was so prolific; Aristotle noted that it brought forth twenty at one birth.

Syphilis attracts the dramatists, both ancient and modern: but with this difference, the old writers looked upon it as deforming the body, the modern writers as deforming the soul. To the modern writers it is a moral catastrophe, to Wycherly and the other seventeenth-century writers it was simply the French, Spanish or Neapolitan itch. To illustrate this difference in outlook let us compare the plots of Brieux's *Damaged Goods*, 1905, and Wycherly's *Country Wife*, 1675.

The plot of *Damaged Goods* is: A syphilitic young man, against doctor's orders, marries; his child is born with congenital lues, a wet nurse is infected, who, finding out the truth, tells the wife and wrecks the home. The play is good theatre and not merely a pamphlet or tract, in spite of its furious indictment against ignorance: ignorance that leaves the young at the mercy of their curiosity and the world in general at the mercy of the people who have the disease and do not realize its danger.

Of course, in quoting from old plays I must use the language of the times, which perhaps seems as coarse to us as it did to the Elizabethan Barber-Surgeon in Middleton's play, *Anything for a Quiet Life ;* who corrects the use of the word pox: "Oh fie! youth, Pox is no word of art: Morbus Gallicus or Morbus Neapolitanus had been well."

*The Country Wife* is a tale of a man who, pretending to have been rendered impotent by the inroads of the pox, gains the tolerance of London's jealous husbands, and gracefully seduces their wives. Wycherly's play was received with enthusiasm. Brieux's play had much the same reception as had Ibsen's *Ghosts.*

When in the late nineteenth century Ibsen began his single-handed crusade against social hypocrisy, the outcry in the London press was hysterical. One great newspaper, for instance, described it as a disgusting representation—as an open drain, a loathsome sore unbandaged. To-day *Ghosts* is recognized both as a stage classic and an effective moral sermon.

Here is the case history of Oswald Alving, the chief character in the play: Mother alive and apparently healthy, father died of syphilis. The young man suffered from headaches when a child. He became an artist and lived in Paris a typical bohemian life. It is made clear in the play that he was not exposed to syphilis. The onset of his illness was at the age of 25, severe headaches, pain in the back of his head, inability to work, loss of energy, and dizzy spells. Two years later he had an attack of short duration which left him "helpless as an infant." It disappeared without sequelae. He consults a Paris specialist who tells him the truth, which he won't believe, his mother having brought him up to idealize his dead father. Mental change and symptoms in Oswald's character are skilfully depicted by Ibsen. Oswald is emotionally unstable, rising from depths of depression to the heights of mania. He is tormented by the thought of becoming helpless and having to be fed with a spoon like a baby, and tries to persuade his mother that when this happens she will poison him with morphia. In the last act Oswald has an attack on the stage and sinks in the chair paralysed and cries: "Give me the Sun, the Sun." If Ibsen meant this for aphasia and that Oswald was calling for the morphia, it is expecting rather a lot from a lay audience.

Now it is interesting that Drs. Esmarch and Jesson in 1857 wrote of the possible relationship between G.P.I. and syphilis, but it was not till 1877 that Dr. Clouston reported a case in a congenital syphilitic. And Oswald's symptoms were very much like the published case of Clouston. In the light of what is known of juvenile paresis Oswald's symptoms, written by a lay person in 1880, are a clever description of this disease.

The first appearance of the pox baffled the medical profession. The treatment at first was sweating and dieting: then from the use of mercury for the itch by the Arabs, mercury was used for syphilis, by fumigation and inunction, and thus the first specific was discovered.

A *double entendre* of the pocky valet Dufroy:—

> "Haste, Dufroy, perform what I commanded you."
> "I vill be very quick, I am more than half de Mercury."
>
> Etherage, *Comical Revenge.*

Under properly regulated mercury treatment the patient spat four pints in twenty-four hours. One method was to place the patient in a tub and have cinnabar thrown on a hot dish in it. The mercury thus volatilized deposited on the surface of the body, and he was then sweated in the tub. (Figures 1, 2.)

The tub used was the one used for curing or corning beef; the beef was well powdered with salt in the process, and thus the tub was called the powdering-tub. This explains Shakespeare's references to the tub:—

*Pompey:* "Troth, Sir, she hath eaten up all her beef, and she is herself in the tub."

*Measure for Measure,* III. ii.

*Pistol:* "To the spital go,
And from the powdering-tub of infamy
Fetch forth the lazar kite of Cressid's kind."

*Henry V,* II. i.

In the lines from Timon of Athens, you will find a really remarkable group of syphilitic symptoms written over three hundred years ago: tibial nodes, laryngeal symptoms, necrosis of nasal bones, alopecia and impotence. Authorities say this is the first published record of the depression of the nose caused by syphilis:—

*Timon:* ". . . Consumptions sow
In hollow bones of man; strike their sharp shins,
And mar men's spurring. Crack the lawyer's voice,
That he may never more false title plead,
Nor sound his quillets shrilly: hoar the flamen,
That scolds against the quality of flesh
And not believes himself: down with the nose,
Down with it flat; take the bridge quite away
Of him that, his particular to foresee,
Smells from the general weal: make curl'd-pate ruffians bald,
And let the unscarr'd braggarts of the war
Derive some pain from you: plague all,
That your activity may defeat and quell
The source of all erection."

*Timon,* IV. iii.

But I suppose this is no worse than Shakespeare trying to make a medico-legal point by throwing doubts upon Joan of Arc's moral character:—

*Joan:* "I am with child, ye bloody homicides:
Murder not then the fruit within my womb,
Although ye hale me to a violent death."

*Henry VI* (2), V. iv.

Showing the frequency of syphilis in those days we have the grave-digger's reply to Hamlet :—

*Hamlet :* " How long will a man lie i' the earth ere he rot ? "

*Grave-digger :* " Faith, if he be not rotten before he die,—as we have many pocky corses nowadays, that will scarce hold the laying in,—he will last you some eight year or nine year."

*Hamlet,* V. i.

And even ladies used the word pox as a kind of oath :—

*Katherine :* " A pox of that jest."

*Love's Labour's Lost,* V. ii.

A connection between gout and the pox was believed in by Doctor Sydenham, who says in his treatise on gout : " Gouty patients are generally rather old men or men who have so worn themselves out in youth as to have brought on premature old age, and of such dissolute habits, none being more common than the premature and excessive indulgence in venery and the like exhausting passions."

This confusion was shown in the seventeenth, century dramas. In fact, the poet Hedylus wrote 300 years B.C. :—

" The daughter of limb-loosening Bacchus and limb-loosening Venus is limb-loosening gout."

" As gout in age from pox in youth proceeds."

Wycherly, *Country Wife.*

" I know whence the pox is now descended : the gout begets it."

Broome, *Damoiselle.*

*Falstaff :* " A pox of this gout ! or, a gout of this pox ! for one or the other plays the rogue with my great toe."

*Henry IV* (2), I. iii.

I should like to give two or three examples of the endeavour of dramatists to keep up to date. In the recent play, *The Amazing Dr. Clitterhouse,* we find a doctor who joins and leads a gang of criminals with the intention of studying the pathology of crime. He believes their experiences have a definite pathological effect on them, and proceeds to investigate on somewhat orthodox lines with blood-pressure examinations, laryngoscopy and blood analyses. The gang accepts with no more protest than "being mucked about every time we does a job; anybody might think we was 'orspital cases." Dr. Clitterhouse taught one of the gang to take blood before and after committing a crime, using a Behring-Venule.

This desire to be up to date is shewn by Shaw in *The Doctor's Dilemma,* written when Wright was working on vaccine therapy. He shews a character suffering from phthisis, hastened to the grave by being given a tuberculin injection when the blood was in the negative phase. The doctor did not believe in having the opsonic index investigated. The play is a splendid skit on our foibles, but loses much of its point by the animus shown by Shaw against our profession. For instance, in his introduction to the play he says: "Surgeons though often more unscrupulous than general practitioners yet retain their self respect more easily."

In the play *Men in White* we have the scene set in a very modern hospital. When a resident or honorary is wanted, loud-speakers in every ward and corridor shout out the name. I am sure our wives would welcome this at the B.R.I. But the most exciting episode is when the heroic young house surgeon dashes the hypodermic syringe from the hand of his chief who insists upon giving insulin to a patient suffering from hypoglycaemia, caused by too much insulin, and thus captures the sympathy of the audience in spite of

### PLATE I

FIG. 1.

Mercurial Fumigation.

FIG. 2.
"The Tub."

*Reproduced from " Eine Sterbende Krankheit," by Dr. Gerh. Venzmer/Montana-Verlag, A.-G., Leipzig.*

PLATE II

Fig. 3.

Uroscopy.

Fig. 4.

Uroscopy. "*Mal d'Amour.*"

having seduced a young probationer: she later dies from septicæmia following an illegal operation performed by an abortionist.

Even in the seventeenth century we find reference soon made in plays to recent discoveries. Cinchona, known as "Jesuit's Powder," was brought to Europe in 1632, and used in England in 1655 by the Jesuits. It was a very highly gratifying experience for a physician of those days to watch the drug cure ague in hours, whereas under older non-specific treatment such as the application of a spider in a nutshell lapped in silk, or the application of chips from a hangman's tree, fevers took weeks to disappear; there were elements of drama in such a contrast. A dramatist in 1679 says:—

> "Marriage is to love as the Jesuit powder is to an ague; it stops the fit and in a little time wears it quite off."
>
> Shadwell, *True Widow.*

Dryden wrote, six years after Harvey's discovery of the circulation of the blood:—

> "The circling streams once thought but pools of blood;
> Whether life's fuel or the bodies food
> From dark oblivion Harvey's name shall save."
>
> Dryden's Introduction to *Choreogigantum* (*i.e.* Stonehenge).
> Walter Charleton. 1668.

In a play a year later, 1669, the keeper of an asylum explains how they whip the inmates out of a frenzy into stark madness and then whip them till they come round to their senses again. On this a character says:—

> "That plainly shews the circulation of the blood."
>
> Lacey, *The Dumb Lady.*

Of course many Bardolaters claim that Shakespeare had anticipated Harvey, quoting to suit their case such lines as these:—

*Brutus :* " You are my true and honourable wife,
As dear to me as are the ruddy drops
That visit my sad heart."

*Julius Cæsar,* II. i.

*Menenius :* " I send it through the rivers of your blood,
Even to the court, the heart."

*Coriolanus,* I. i.

But we might as well say that he anticipated the treatment of G.P.I. by malaria :—

*Benvolio :* " Take thou some new infection to thy eye
And the rank poison of the old will die."

*Romeo,* I. ii.

or anticipated Jazz :—

*Richard :* " The music mads me, let it sound no more. . . .
In me it seems it will make wise men mad."

*Richard* II, V. V.

or our new threepenny pieces :—

*Hamlet :* " Thou comes't in such a questionable shape."

*Hamlet,* I. V.

We can, however, take the credit given by the sensational press in the silly season of last year to a Dr. Clifford Allen, for a supposed original discovery that fat men were less neurotic and more dependable in emergencies than thin men, and give the credit to Shakespeare :—

*Caesar :* " Let me have men about me that are fat ;
Sleek-headed men and such as sleep o' nights.
Yond Cassius has a lean and hungry look ;
He thinks too much : Such men are dangerous."

*Julius Cæsar,* I. ii.

There is no doubt that if we allow our idolatry to get the better of our sense of proportion, we could say that Tennyson in *Maud* anticipated William Meyer's description of the adenoid facies : " A rabbit mouth that is ever agape."

Sir St. Clair Thomson thought that Shakespeare had described singers' nodules :—

> *Falstaff :* "For my voice, I have lost it with hollaing, and singing of anthems."
>
> *Henry IV* (2), I. ii.

but when he discovered that Sir John Falstaff had been exposed to syphilis and addicted to indulgence in wine, he thought it more likely tertiary syphilis in an alcoholic subject.

The historian Green says : "The earliest dramatists were for the most part poor and reckless in their poverty, wild livers, defiant of law and common fame, in revolt against the usages and religion of their day : atheists in general repute, 'holding Moses for a juggler,' haunting the brothel and the alehouse and dying starved or in tavern brawls." It is thought they obtained their knowledge of medicine in wandering systematically from the alehouse to the Hall of Barber Surgeons.

Whatever may have been their method, it is certain that the dramatists display an acquaintance with medicine so unusual and extensive that it must have been level with the highest knowledge of their time. As is shewn by their talk of pericranium, brain-pan, pia mater, cerebrum, cutis, spinal medulla and the colon : "The colon of the gentleman should be filled with answerable food."—Middleton, *The Chaste Maid of Cheapside.*

One form of quackery, the pretensions of which were seen through by the seventeenth-century dramatists, was water-casting, and some great artists have illustrated the procedure. (Figures 3, 4.) Uroscopy or water-casting is said to have arisen from the ecclesiastical interdicts placed upon the medical vocations of the clergy. Priests and monks not allowed to visit their former patients are said to have resorted to diagnosing their cases and directing their treatment upon a simple inspection of the urine. Apparently the custom was not limited to the priesthood, for we find this line :—

> *Fabian:* " Carry his water to the wise woman."
>
> *Twelfth Night,* III. iv.

This indicated female quacks in the business. That some doctors were not beyond reproach in the matter is suggested by an old statute of the Royal College of Physicians, in which the members were forbidden to give advice upon the mere inspection of urine. The sixteenth-century dramatists made several references to this subject :—

> " Tell but some women a secret overnight, your doctor may find it in the urinal in the morning."
>
> Tourneur, *Revenger's Tragedy.*

Nowadays to detect pregnancy I believe the woman's urine is injected into immature rabbits. In the seventeenth century the procedure was simpler :

> " I was once sick and I took my water in a basket and carried it to a doctor."
> " In a basket ? "
> " Yes, sir, you arrant fool, there was a urinal in it."
> " I cry for mercy."
> " The doctor told me I was with child."
>
> Dekker and Webster, *Northward Ho.*

But as with the more elaborate tests of to-day the uroscopists some times failed. The doctor in another play diagnosed pregnancy by uroscopy, but at full time no baby had arrived.

> " What had she then ? "
> " Only a fit of the mother."
> " It's possible our doctor's urinal judgment is half cracked then."
>
> Jonson, *Magnetic Lady.*

"A fit of the mother" is a name for hysteria:—

> "Then for his person 'tis comparably odious, he hath such a breath, one kiss of him were enough to cure the fits of the mother. 'Tis worse than asafoetida."
>
> Otway, *Soldier of Fortune.*

Congreve also speaks of asafoetida as a cure for hysteria:—

> "Is there a worse disease than the conversation of fools?"
> "Yes, the vapours: fools are physic for it next to asafoetida."
>
> Congreve, *Way of the World.*

Uroscopy or water-casting is used in Macbeth to contribute a most effective metaphor:—

> *Macbeth:* "If thou could'st, doctor, cast
> The water of my land, find her disease,
> And purge it to a sound and pristine health,
> I would applaud thee to the very echo,
> That would applaud again."
>
> *Macbeth,* v. iii.

Speaking of medical metaphors, here is one that shows that Cæsarean section must have been well known to the audience of those days:—

> *Bastard:* "You bloody Neroes, ripping up the womb
> Of your dear mother England, blush for shame."
>
> *King John,* v. ii.

and of the infectious nature of disease:—

> *Falstaff:* "It is certain that either wise bearing or ignorant carriage is caught, as men take diseases, one of another; therefore let men take heed of their company."
>
> *Henry IV* (2), v. i.

Uroscopy began gradually to lose its hold in the seventeenth century:—

> "There is more credit to be given to the face, than to a sick man's urine, which some call the physician's whore, because she cozens him."
>
> Webster, *Duchess of Malfi.*

I don't think modern urine analysis will make the same appeal to twentieth-century dramatists.

URINE EXAMINATION.

| | |
|---|---|
| Reaction .pH. .. .. | 7 |
| Specific Gravity .. | 1018 |
| Albumen .. .. | Nil |
| Blood .. .. | Nil |
| Sugar .. .. .. | Nil |
| Urea .. .. .. | 1·3 |
| Acetone Bodies .. | Nil |
| Bilirubin .. .. | Nil |
| Chorides .. .. | 1% |
| Indican .. .. | Absent |
| Bence-Jones Protein .. | Absent |
| Diastase .. .. | 20 |
| Hormones .. .. | Negative |
| Deposit .. | Nil abnormal |
| Culture .. .. | Sterile |

This looks impressive and we general practitioners look duly impressed when we read it: but when is added a diagnostic and prognostic conclusion, do we really pay enough attention to the fact that the person who has done it for us is not of necessity a doctor and at any rate has never seen the patient? Isn't it reminiscent of the conversation between Falstaff and his page :—

> *Falstaff:* "Sirrah, you giant, what says the Doctor to my water?"
>
> *Page:* "He said, Sir, the water itself was a good healthy water; but for the party that owed it, he might have more diseases than he knew for."
>
> *Henry IV* (2), I. ii.

While on the subject of urine, have you ever heard of bagpipes causing enuresis ?—

> "What ails thy brother, cannot he hold his water at the reading of a ballad?"
> "Oh no, a rhyme to him is worse than cheese or a bagpipe."
>
> Jonson, *Every Man in his Humour.*

> *Shylock:* "Some men there are like not a gaping pig,
> Some that are mad if they behold a cat,
> And others, when the bagpipe sings i' the nose,
> Cannot contain their urine."
>
> *Merchant of Venice,* III. v.

Weir Mitchell was interested in collecting examples of the psychasthenic symptom of fear of cats, observed and recorded by Shakespeare centuries ago.

One can only deal very briefly with the extraordinary insight Shakespeare had of psychology and of mental diseases. First the suggested treatment of madness, not adopted till our time.*

> *Leonato :* " Fetter strong madness in a silken thread,
> Charm ache with air, and agony with words."
>
> *Much Ado,* v. i.

While the treatment of the time is recorded in *As You Like It* :—

> *Rosalind :* " Love is merely a madness ; and, I tell you, deserves as well a dark house and a whip as madmen do."
>
> *As You Like It,* III. ii.

A modern neurologist has said that it is possible to detect epileptics by their faces alone :—

> *Kent :* " A plague upon your epileptic visage."
>
> *King Lear,* II. ii.

In spite of the extravagant, unintelligible and unpronouncable nomenclature of the mental specialists of to-day, have we advanced on Polonius's definition of insanity ?

> *Polonius :* " Your noble son is mad :
> Mad call I it ; for, to define true madness,
> What is't but to be nothing else but mad ? "
>
> *Hamlet* III. ii.

By the way, I don't think Shakespeare, in spite of his psychological knowledge, would have been a disciple of Freud :—

> *Bottom :* " I have had a dream, past the wit of man to say what dream it was : Man is but an ass, if he go about to expound this dream."
>
> *A Midsummer Night's Dream,* IV. i.

* Leonato uses these words to illustrate *impossibilities.*—ED.

Here are two three-to-four-hundred-years-old extracts dealing with the bad psychological effect of repression, a term so beloved of our modern psychologists :—

> " He oft finds medicine who his grief imparts,
> But double griefs affect concealing hearts."
>
> Spenser, *Faery Queen.*

> " Those are the killing griefs which dare not speak."
>
> Webster, *White Devil.*

Chlorosis, or green-sickness, a disease which was formerly common, but now seems to have disappeared, was used as a term of abuse :—

> *Capulet :* " Out, you green - sickness carrion ! Out, you baggage !
> You tallow face !
>
> *Romeo,* III. V.

It was also known that men could suffer from it :—

> *Falstaff :* " Good faith, this same young sober-blooded boy doth not love me ; nor a man cannot make him laugh ; but that's no marvel, he drinks no wine. There's never any of these demure boys come to any proof ; for thin drink doth so over-cool their blood, and making many fish-meals, that they fall into a kind of male green-sickness ; and then, when they marry, they get wenches."
>
> *Henry IV* (2), IV. iii.

We have no evidence, however, to support Shakespeare's idea that either alcohol or male green-sickness will influence the sex of the offspring. Perhaps Falstaff was only using the term as one of derision, equivalent to our present day term of " cissy."

The general opinion is that operations under general anæsthesia date from the discovery of chloroform and ether : " He is a clever operator is Walpole, but he's only one of your chloroform surgeons. In my early days you made your man drunk and the porters and students held him down and you had to set your teeth and finish the job fast." (Shaw, *Doctor's Dilemma.*) But in 1632 we find these words :—

"I'll imitate the pities of old surgeons
To this lost limb, who ere they show their art,
Cast one asleep, then cut the diseased part."

Middleton, *Women Beware Women.*

Talking of drugs, the most commonly spoken of by the old dramatists was rhubarb : "I say that all these doctors 'ill do her no more good than a swig of water, your daughter wants summat else than rhubarb, a husband's a plaster that cures all lasses' complaints" (Molière, *Médecin malgrè lui*). I suppose aspirin is the one drug you would hear spoken of most in modern plays.

Shakespeare mentions aphrodisiacs :—

*Falstaff :* "Let the sky . . . snow eringoes."

*Merry Wives of Windsor,* v. iv.

The eringo root (sea holly) had some fame as an aphrodisiac perhaps because of its shape, and Dr. Bucknill reports that Dr. Hall, Shakespeare's son-in-law, prescribed it for his own wife. Dryden mentions camphor as an anaphrodisiac :—

"But of this Jezebel of mine, I'll get a physician that shall prescribe her an ounce of camphor every morning for her breakfast to abate incontinency."

Dryden, *Spanish Friar.*

I like best the references to the remedies our mothers still prescribe :—

*Bottom :* "I shall desire you of more acquaintance, good master Cobweb : if I cut my finger, I shall make bold with you."

*A Midsummer Night's Dream,* III. i.

"Let me see your arm, Sir, I must have some powdered sugar to stop the blood."

*Beau Stratagem.*

> "Fie, fie, what's all this. One of your eyes bloodshot ?
> Use my ring to it, they say 'tis very sovereign."
>
> Webster, *Duchess of Malfi.*

Shakespeare sometimes uses the word medicine as meaning a doctor :—

> *Lafeu :* "I have seen a medicine
> That's able to breathe life into a stone,
> Quicken a rock, and make you dance canary
> With sprightly fire and motion."
>
> *All's Well That Ends Well,* II. i.

The medicine referred to is a lady doctor. And if we may judge the lady doctors of Shakespeare's time by our own charming colleagues, the whole thing is understandable.

An American writer, Charles Dana, says : "Medical satire and dispraise through the ages have been always much of the same kind and all the real solid and elemental jests against doctors were uttered over 2,000 years ago, and are being served up in the language of our times." Here is one that every generation serves up, taken from a seventeenth-century play :—

> "You make no more haste than a physician to a patient that has no money."
>
> Jonson, *Poetaster.*

Talking of fees, the recognized fee in Elizabethan times was an angel, about 10s. of our money. This led Culpeper to write :—

> "Physicians of the present day are like Balaam's ass, they will not speak until they see an angel."

Molière in the seventeenth century was witty and satirical in his plays at our expense, but his plays do not lend themselves well to brief quotation, and besides, they are well known. Marocreau de Brècourt wrote a farce soon after Molière's death showing Molière in

Hades defending himself against four doctors who had also arrived there. These are his words :—

> "It is not against medicine that I have so inveighed. I have adored the healing art, I revere its judicious practices, but I abhor and detest the pernicious and wicked use made of it by senseless fools whom only a diploma makes doctors. You perpetually prove your ignorance and uncertainty by constant disagreements. Never in your simplest prescriptions has the world ever seen you follow each other's prescriptions without adding or subtracting something. As to your opinions they are more varied than your practice. . . . You take every advantage of mankind's weakness and superstition, and in this dangerous moment you brazenly experiment with our lives with any remedy that may suggest itself to your ambitious imagination. You try anything in this confidence, that the sun shines on your successes and the earth hides all your failures.
>
> De Brècourt, *The Ghost of Molière.*

Did not Lloyd George, perhaps not a dramatist but rather a dramatic personage, echo Molière's words three hundred years afterwards, when he said of doctors ? "I found them unruly. The doctors are always changing their opinion. They always find some new fad."

I should like to ask if you agree with Sir Robert Hutchison, who says that from the middle of the nineteenth century down to the present time the progress of medicine has been so remarkable and its benefits to the community so conspicuous that the old jibes at doctors have lost their point, and are only repeated in jest.

I think the Americanism "sez you" would be the answer of most of us. At any rate the dramatists give us credit of having the decency not to talk about our patients :—

> *Surgeon :* "When we cure gentlemen of foul diseases, they give as much for the cure and twice as much that we do not blab on't."
>
> Webster, *Devil's Law Case.*

"You must be secret as your midwife or a barber surgeon."
Dekker, *Honest Whore.*

"I'll bring half the chirurgeons in town to swear it."
"They, they'll swear a man, bled to death through his wounds, died of apoplexy."
Wycherly, *Country Wife.*

V. B. Green Armitage has written a lengthy paper dealing only with his own speciality and Shakespeare. Here is his reference to longing of pregnancy :—

*Pompey :* "Sir, she came in, great with child, and longing, —saving your honour's reverence,—for stewed prunes."
*Measure for Measure,* II. i.

I should have liked to say something of the old Mummers' plays, all of which contain amusing medical dialogue, but I do hope my ineptitude will not put you off the subject.

May I end with three quotations from England's greatest dramatist, which, though written over 300 years ago, seem to me to be as applicable to our profession now as then ?

*Cleopatra :* "Though it be honest, it is never good
To bring bad news."
*Antony and Cleopatra,* II. v.

*Rosalind :* "Your task shall be,
With all the fierce endeavour of your wit
To enforce the pained impotent to smile."
*Love's Labour's Lost,* V. ii.

*Claudio :* "The miserable have no other medicine
But only hope."
*Measure for Measure,* III. i.

---

LADIES AND GENTLEMEN,*

I was greatly flattered when asked to read to the Galenicals the Presidential Address I had given to the Medico-Chirurgical Society. The first thing I did was

* This portion was added as an introduction to an address to "The Galenicals."

to consult a dictionary to find out what the word Galenicals meant. I found that it was a term used to denote Standard Preparations, and I found that "standard" meant a degree of excellence. So you can imagine how honoured I was, and yet how diffident I feel when addressing a society composed of members having reached a degree of excellence.

I had thought it meant you were disciples of Galen: that huge, overshadowing figure, which eclipsed every other luminary in the playwright's mind, even that of Hippocrates himself. His name sanctioned any medical custom and sanctified every medical act:—

> "Arise, arise, your breakfast stays,
> Good water gruel warm
> Or sugar sops which Galen says
> With maice will do no harm."
>
> *Davenant.*

> "For I observe, so soon as his searching eyes had fastened upon her, his labouring pulse, that, through his fever, did before stick hard and frequent, now exceeds in both these differences; and this Galen himself found true upon a woman that had doted upon a dancer."
>
> John Shirley, 1628, *The Witty Fair One.*

Psychiatrists can claim Galen as one of the first luminaries of their art. The case, to which the pretended doctor in this play refers, illustrates the ingenuity and penetration of a first class physician. Galen, as he tells in his book on prognosis, had been called in to see a woman who was stated to be sleepless at night. These are his words:—

> "After I had diagnosed that there was no bodily trouble, and that the woman was suffering from some mental uneasiness, it happened, that at the very time I was examining her this was confirmed. Somebody came from the theatre and said he had seen Pylades dancing. Then both her expression and the colour of her face changed. Seeing this, I applied my hand to her wrist, and noticed that her pulse had suddenly become extremely irregular. So on the next day I said to one of my followers that when I paid my visit he was to announce another dancer. When he did this I found the pulse was unaffected. He again did it with another name, and again the pulse was unaffected. On the fourth night, Pylades was again announced, and again the pulse was affected. Then I found out that the woman was in love with Pylades."

Before reading my paper I should like to give a few extra references from plays that I thought would be of interest to the students.

Ladies first: Cosmetics:—

"Shall we protest to the ladies that this painting makes them angels? No, Sir. Such vices as stand not accountable to law should be cured as men cure tetters, by casting ink upon them."

Marston, *Malcontents*, 1604.

"There's knavery in daubing."

Dekker, *Honest Whore.*

*Hamlet:* "I have heard of your paintings, too, well enough; God hath given you one face, and you make yourselves another."

*Hamlet*, III. i.

"Thou most ill-shrouded rottenness, thou piece made by a painter and apothecary."

Beaumont and Fletcher, *Philaster*, II. iv.

And now Gentlemen. I have been impressed by your lack of descriptive power in taking notes: you have Shakespeare's authority for its usefulness.

*Doctor:* "I will set down what comes from her to satisfy my remembrance of her."

*Macbeth*, V. i.

Your imagination does not seem to go beyond a "bag of worms." Now listen to Shakespeare's description of a mole:—

*Iachimo:* " On her left breast
A mole, cinque-spotted, like the crimson drops
I' the bottom of a cowslip."

*Cymbeline*, II. ii.

I am sure your teachers would appreciate your notes if written like this.

Ladies and Gentlemen: Love :—

" Oh ye Gods, have ye ordained for every malady a medicine, for every sore a slave, for every pain a plaster, leaving only love remediless."

Lyly, *Euphues.*

In another play of the same period, a physician says :—

" . . . You are in love."
" I think I am. What's your appliance now ?
Can all your Paracelsian mixtures cure it."

Middleton and Rowley, *Fair Quarell.*

Don't be despondent, it is not hopeless. Dryden says :—

" And love may be expelled by other love,
As poisons are by poisons."

Dryden, *All for Love.*

### BIBLIOGRAPHY.

Excerpts from several of the publications listed below have been incorporated in this paper, and their use is hereby acknowledged :—

Cumberland Clark, *Shakespeare and Psychology.*

Sir St. Clair Thomson, *Shakespeare and Medicine, Trans. Med. Soc. Lond.*, 1916, xxxix. 257.

Harold Bailey, *Shakespeare Symphony.*

Bayer's *Clinical Excerpts.*

V. B. Green Armitage, *Surgery, Gynæcology and Obstetrics*, 1930.

V. B. Green Armitage, *Indian Medical Gazette*, 1930.

Witkoski, *The Evil that has been said of Doctors.*

L. M. Griffiths, *Bristol Medico-Chirurgical Journal*, 1887, v. 225.

Annals of Medical History :—
Fowler, *Chlorosis, an Obituary.*
Silvette, *Doctors on the Stage.*
Klauder, *Syphilis and the Characters in Ibsen's Dramas.*
Watson, *Medical Lore in Shakespeare.*

*Three Plays by Brieux* (Cape).

McLeod Yearsley, *Doctors in Elizabethan Drama.*

Moyes, *Medicine in Plays of Shakespeare.*

Gerhard Venzmer, *Eine Sterbende Krankheit* (Bayers).

## Figures and Tables

**Fig. 1. Fig. 2. f1:**
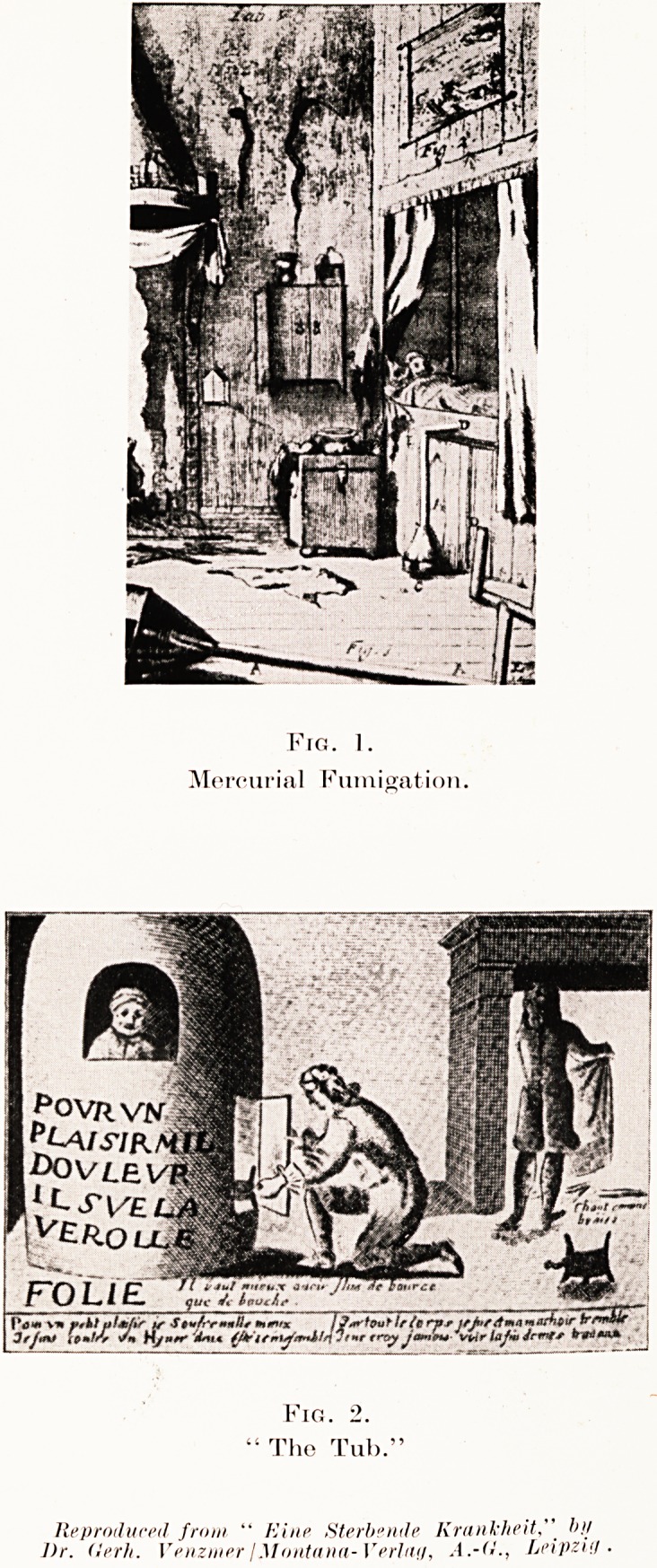


**Fig. 3. f2:**
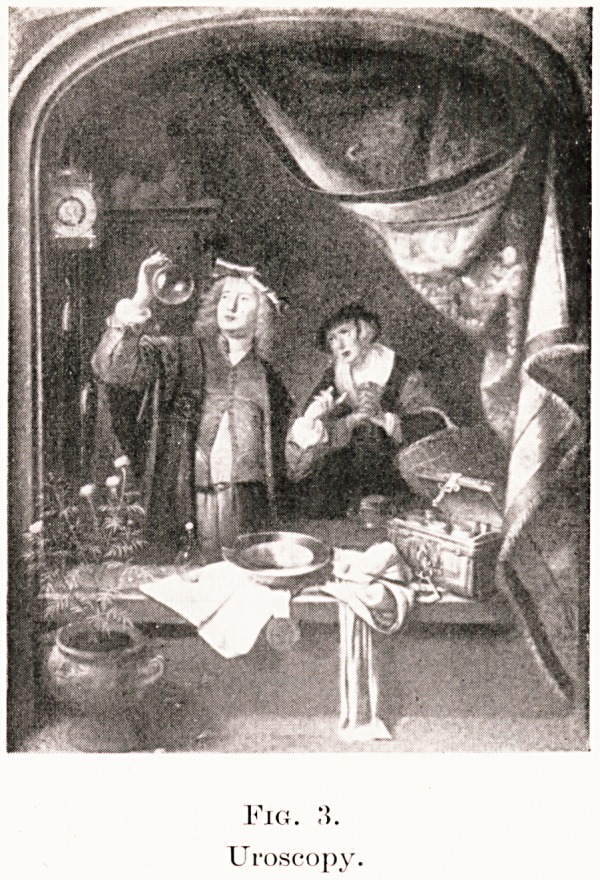


**Fig. 4. f3:**